# Supplementary material for: Algorithms for the selection of fluorescent reporters
Source: Commun Biol. 2021 Jan 26;4:118. doi: 10.1038/s42003-020-01599-5 (PMC7838271; doi:10.1038/s42003-020-01599-5)
Supplement: Supplementary file 3 — Description of Additional Supplementary Files [file 42003_2020_1599_MOESM3_ESM.pdf]

Description of Additional Supplementary Files

File Name: Supplementary Data 1

Description: A CSV containing the normalized values of the signal and bleedthrough for each detector of the 10-color panel solution in the case study.

File Name: Supplementary Data 2

Description: A zipped file containing 3 CSVs, where each CSV is the configuration of the measurement instruments used in the case study.

File Name: Supplementary Data 3

Description: A zipped file containing 3 CSVs, where 2 CSVs contain the fluorophore spectra of the fluorophores used in the case study and 1 CSV contains the brightness of the fluorophores used in the case study.

File Name: Supplementary Data 4

Description: A zipped file containing CSVs and Excel files that contain the data for the plots and figures in the main text.
