# Supplementary material for: Algorithms for the selection of fluorescent reporters
Source: Commun Biol. 2021 Jan 26;4:118. doi: 10.1038/s42003-020-01599-5 (PMC7838271; doi:10.1038/s42003-020-01599-5)
Supplement: Supplementary file 8 — Reporting Summary [file 42003_2020_1599_MOESM8_ESM.pdf]

nature research

Corresponding author(s): Douglas Densmore, PhD

Last updated by author(s): Nov 5, 2020

Reporting Summary

Nature Research wishes to improve the reproducibility of the work that we publish. This form provides structure for consistency and transparency in reporting. For further information on Nature Research policies, see our Editorial Policies and the Editorial Policy Checklist.

Statistics

For all statistical analyses, confirm that the following items are present in the figure legend, table legend, main text, or Methods section.

n/a	Confirmed	
x		The exact sample size (*n*) for each experimental group/condition, given as a discrete number and unit of measurement
x		A statement on whether measurements were taken from distinct samples or whether the same sample was measured repeatedly
x		The statistical test(s) used AND whether they are one- or two-sided<br>*Only common tests should be described solely by name; describe more complex techniques in the Methods section.*
x		A description of all covariates tested
x		A description of any assumptions or corrections, such as tests of normality and adjustment for multiple comparisons
	x	A full description of the statistical parameters including central tendency (e.g. means) or other basic estimates (e.g. regression coefficient) AND variation (e.g. standard deviation) or associated estimates of uncertainty (e.g. confidence intervals)
x		For null hypothesis testing, the test statistic (e.g. *F*, *t*, *r*) with confidence intervals, effect sizes, degrees of freedom and *P* value noted<br>*Give P values as exact values whenever suitable.*
x		For Bayesian analysis, information on the choice of priors and Markov chain Monte Carlo settings
	x	For hierarchical and complex designs, identification of the appropriate level for tests and full reporting of outcomes
x		Estimates of effect sizes (e.g. Cohen's *d*, Pearson's *r*), indicating how they were calculated

Our web collection on statistics for biologists contains articles on many of the points above.

Software and code

Policy information about availability of computer code

Data collection	Experimental data was saved as FCS3.0 files and was analyzed using R Bioconductor packages
Data analysis	The scripts to analyze and compare the experimental and computational results as well as the scripts used to generate the images are all included in the open-source github repository (https://github.com/CIDARLAB/fpSelection).

For manuscripts utilizing custom algorithms or software that are central to the research but not yet described in published literature, software must be made available to editors and reviewers. We strongly encourage code deposition in a community repository (e.g. GitHub). See the Nature Research guidelines for submitting code & software for further information.

Data

Policy information about availability of data

All manuscripts must include a data availability statement. This statement should provide the following information, where applicable:

- Accession codes, unique identifiers, or web links for publicly available datasets
- A list of figures that have associated raw data
- A description of any restrictions on data availability

The data used in this manuscript will be available in the supplemental information as well as the github repository (https://github.com/CIDARLAB/fpSelection)

Field-specific reporting

Life sciences study design

All studies must disclose on these points even when the disclosure is negative.

Sample size	All cell lines expressing fluorescent proteins were grown and measured as biological triplicates
Data exclusions	No data exclusions applied
Replication	All data presented is backed by three biological replicates
Randomization	N/A
Blinding	N/A

Reporting for specific materials, systems and methods

We require information from authors about some types of materials, experimental systems and methods used in many studies. Here, indicate whether each material, system or method listed is relevant to your study. If you are not sure if a list item applies to your research, read the appropriate section before selecting a response.

Materials & experimental systems

n/a	Involved in the study
☒	☐ Antibodies
☐	☒ Eukaryotic cell lines
☒	☐ Palaeontology and archaeology
☒	☐ Animals and other organisms
☒	☐ Human research participants
☒	☐ Clinical data
☒	☐ Dual use research of concern

Methods

n/a	Involved in the study
☒	☐ ChIP-seq
☐	☒ Flow cytometry
☒	☐ MRI-based neuroimaging

Eukaryotic cell lines

Policy information about cell lines

Cell line source(s)	PGP1 iPS cell line provided by the Personal Genome Project at Harvard University
Authentication	Cell lines authenticated before initiation of this study
Mycoplasma contamination	Cell lines were only used as a chassis for expressing fluorescent proteins and therefore no underlying biological insights were derived from this study and therefore it did not appear relevant to test every cell line for mycoplasma. However, the base cell line used to make these cell lines was validated to be free of mycoplasma contamination.
Commonly misidentified lines (See ICLAC register)	N/A

Flow Cytometry

Plots

Confirm that:

☒ The axis labels state the marker and fluorochrome used (e.g. CD4-FITC).

☒ The axis scales are clearly visible. Include numbers along axes only for bottom left plot of group (a 'group' is an analysis of identical markers).

☒ All plots are contour plots with outliers or pseudocolor plots.

☒ A numerical value for number of cells or percentage (with statistics) is provided.

Methodology

Sample preparation	Cells lines with a tet-inducible fluorescent protein cassettes were created prior to the study and selected for with puromycin. When acquiring measurements, cell lines were grown to confluency and fluorescent protein expression was induced for 6 days prior to harvesting and measurement. Cells were imaged after fixation and resuspension in PBS
Instrument	BD LSRFortessa, MACSQuant, and CytoFLEX

Software	Data saved as FCS3.0 files and were analyzed using R Bioconductor packages
Cell population abundance	At least 10,000 cells were measured per individual sample
Gating strategy	Cells not gated

☐ Tick this box to confirm that a figure exemplifying the gating strategy is provided in the Supplementary Information.
